# High Capacity for Physiological Plasticity Occurs at a Slow Rate in Ectotherms

**DOI:** 10.1111/ele.70046

**Published:** 2025-01-20

**Authors:** Tim Burton, Sigurd Einum

**Affiliations:** ^1^ Norwegian Institute for Nature Research Trondheim Norway; ^2^ Department of Biology, Centre for Biodiversity Dynamics Norwegian University of Science and Technology Trondheim Norway

**Keywords:** acclimation capacity, acclimation rate, acclimation response ratio, CTmax, CTmin, phenotypic plasticity, rate of plasticity, reaction norm, thermal tolerance, timescale of plasticity

## Abstract

Phenotypic plasticity enables organisms to express a phenotype that is optimal in their current environment. The ability of organisms to obtain the optimum phenotype is determined by their (i) capacity for plasticity, which facilitates phenotypic adjustment corresponding to the amplitude of environmental change but also their (ii) rate of plasticity, because this determines if the expressed phenotype lags behind changes in the optimum. How the rate of‐ and capacity for plasticity have co‐evolved will thus be critical for the resilience of organisms under different patterns of environmental change. To evaluate the direction of the evolved relationship between plasticity rate and capacity, we reanalysed experiments documenting the time course of thermal tolerance acclimation to temperature change across species of ectothermic animals. We found that the rate and capacity with which thermal tolerance responds plastically to temperature change are negatively correlated, a pattern inconsistent with current theory regarding the evolution of phenotypic plasticity.

## Graphical abstract

The amount or capacity with which phenotypic plasticity occurs helps organisms obtain a phenotype that is optimal for their current environment. However, phenotypic plasticity may not yield a fitness payoff if it lags behind the local optimum. We reanalyse published data, showing that the rate and the capacity with which physiological tolerance can respond plastically to temperature change is negatively correlated across species of cold‐blooded animals. This pattern suggests that the rate of‐ and capacity for phenotypic plasticity have co‐evolved in response to different patterns of environmental variation.

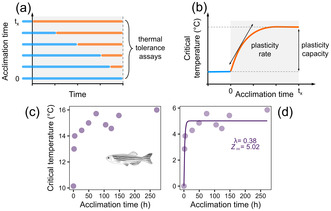

## 1 Introduction

Within their lives organisms experience environments that change by different amplitudes and at different rates, meaning there will be corresponding shifts in the phenotype that maximises fitness (i.e., optimal phenotype). Organisms have evolved different strategies to track such change in the optimal phenotype with the evolved strategy being expected to depend on the timescale of environmental change (relative to the generation time) and on the degree of predictability in the environment (Botero et al. 2015). When environmental change is relatively rapid but also predictable, phenotypic plasticity is expected to be the primary mode of response because it can facilitate an adaptive match between the organism phenotype and the phenotype that is optimal in the current environment (Padilla and Adolph 1996; Gabriel 2005; Gabriel et al. 2005; Siljestam and Östman 2017, but see Ghalambor et al. 2007 for discussion regarding nonadaptive outcomes of phenotypic plasticity). Hence, the fitness payoff arising from phenotypic plasticity will depend simultaneously on *how much* and *how rapidly* plasticity can facilitate the required shift in phenotype. Such plastic shifts in phenotype may be irreversible, as in the case of developmental plasticity (e.g., the production of protective armour by zooplankton in response to the threat of predation, Tollrian 1990) or occur repeatedly and reversibly throughout life as described by reversible plasticity (e.g., the adjustment of photosynthesis in response to changes in light intensity, Bazzaz and Carlson 1982). By far, the most studied aspect of phenotypic plasticity is the amount by which the phenotype can be changed (hereafter the ‘capacity for plasticity’, Lande 2009, 2014; Seebacher, White, and Franklin 2015; Morgan et al. 2022; Pottier et al. 2022). The capacity for plasticity describes the change in phenotype per unit change in the environment (or reaction norm slope, Schlichting and Pigliucci 1998). The deviation between capacity and the change in the optimal phenotype across environments determines the extent of matching between the expressed phenotype and environment once the plastic response has been established. In comparison with this focus on plasticity capacity, little heed has been paid to the rate at which phenotypic plasticity occurs (Burton, Ratikainen, and Einum 2022; Dupont et al. 2024). However, the rate of plasticity will also influence the fitness payoff resulting from the match between the realised phenotype and the environment by determining the extent to which the change in the phenotype lags behind the change in the environment and optimal phenotype (Fey et al. 2021). Thus, throughout evolution, it seems probable that patterns of environmental fluctuations have likely shaped variation and covariation in both the rate and capacity of phenotypic plasticity.

We recently revealed systematic variation in the rate of phenotypic plasticity in thermal tolerance across groups of ectothermic animals, with rates of plasticity being most rapid in amphibians and reptiles, intermediate in insects and slowest in crustaceans and fishes (Einum and Burton 2023). If these differences are not the result of random drift, it suggests that the rate component of phenotypic plasticity might also evolve in response to selection, an outcome not considered explicitly in current theory (Siljestam and Östman 2017; Burton, Ratikainen, and Einum 2022). Given this apparent evolution of plasticity rate (Einum and Burton 2023), it begs the question of whether there has been a corresponding evolutionary shift in the capacity of plasticity as the direction of any covariation between plasticity rate and capacity may be critical for the ability of organisms to optimise their phenotypes in environments that fluctuate. Specific predictions regarding any directionality of the relationship between plasticity rate and plasticity capacity are lacking in the literature. However, some insight can be gleaned from current theory. A key conclusion of quantitative modelling is that fluctuations in the environment must be sufficiently predictable for plasticity to evolve (Lande 2009, 2014; Ezard, Prizak, and Hoyle 2014; Tufto 2015). Yet precisely how the rate of plasticity is also expected to evolve under different patterns of fluctuation in the environment remains obscure. Both Siljestam and Östman (2017) and Lande (2014) provide models that can be interpreted to predict a positive relationship between the rate and capacity of plasticity. For example, in Lande's work (2014), environmental predictability, when averaged over development time, increases with the rate of plasticity. In other words, more rapidly plastic organisms should be able to more accurately ‘predict’ the future selective environment, resulting in selection for a greater capacity for plasticity (i.e., steeper reaction norm). However, the main focus of both Siljestam and Östman (2017) and Lande (2014) is to make predictions regarding the evolution of plasticity capacity. Neither consider scenarios where plasticity rate can also evolve in response to the pattern of environmental fluctuation. Other possibilities also exist regarding directionality in the relationship between the respective components of plasticity. There may be no relationship. Such a pattern could arise if, for example, the costs associated with maintaining the ability to be rapidly plastic are negligible meaning that organisms are able to maximise the speed of their plasticity responses irrespective of how large they might be. Alternatively, plasticity rate and capacity could covary negatively if there is a trade‐off in the allocation of resources required for maintaining or producing the rate and capacity of a given plasticity response, or if plasticity responses of large capacity simply take more time to produce.

Our aim was to test for a correlation between the rate and capacity of plasticity at a species level, thus providing key insight into the question of how these traits are related, and to evaluate whether there is empirical support for or against the possibility that the rate of plasticity also evolves in response to the pattern of environmental fluctuations. To test for a correlation between the rate and capacity of plasticity, we drew upon an existing database of experiments describing the acclimation time course of whole‐body thermal tolerance to temperature change (Einum and Burton 2023). In these experiments, the study animals (either reptiles, amphibians, fish, insects, flatworms or crustaceans), previously acclimated to a starting temperature, were abruptly shifted to a new cooler or warmer acclimation temperature. Thermal tolerance was measured prior to and at different time steps after this shift. Measurements of thermal tolerance, which reflect the ability of organisms to deal with stress imposed by temperature (Gunderson, Dillon, and Stillman 2017; Morley et al. 2019), typically display a reversible plastic response to acclimation temperature (Gunderson and Stillman 2015). Plasticity in thermal tolerance might therefore provide an important means for populations to cope with suboptimal temperature exposure, potentially buying time for slower evolutionary responses (Diamond and Martin 2021). Thus, we assume that plasticity in thermal tolerance is adaptive. We implement a novel method to simultaneously estimate the rate and capacity of plasticity from the experimental data and apply meta‐analytical models to extract species‐specific estimates. Across these broad groupings of ectothermic animals, we observed a negative relationship between the rate and the capacity with which the phenotype can be adjusted by plasticity.

## 2 Materials and Methods

### 2.1 Data Collection

We utilised the existing database of plasticity time course experiments provided by Einum and Burton (2023). This database contained data from 308 experiments from 60 studies. The procedure for compiling studies, eligibility criteria for inclusion and the method applied for estimating rates of plasticity is given in detail in (Einum and Burton 2023). Briefly, though, these studies consisted of experiments where animals, having been acclimated to a given initial temperature, were abruptly shifted to a new acclimation temperature (either lower or higher). Thermal tolerance was measured prior to and at different time steps t (in h) after this shift. Measures of thermal tolerance were either critical maximum temperature (CTmax) or critical minimum temperature (CTmin). The data set also contained a lower number of experiments that had measured the time to some defined response such as immobility or death when exposed to a detrimental high or low temperature (TTD). However, for the current work, TTD measurements were excluded, as the approach we used estimated plasticity capacity on the original scale of the data (see below), and thus these were not comparable for measurements of critical temperatures and TTD. This left data from 277 experiments from 47 studies. The model fitted to each data set (see below) estimates two parameters, and thus to avoid overparameterisation we excluded 36 experiments with less than three time points of observation following the initial value measured at time = 0. Finally, we removed another 36 experiments that were missing acclimation temperatures (required for calculating capacity, see below). Thus, the data set available for estimation of rate and capacity consisted of 205 experiments originating from 39 studies. For each experiment, a time series of phenotypic values, *z*_*t*_, charting the time course of the acclimation response were obtained, typically from figures showing the mean tolerance of groups of individuals that had experienced the ‘new’ temperature for differing lengths of time.

### 2.2 Estimating the Rate Of‐ and Capacity for Phenotypic Plasticity

**FIGURE 1:**
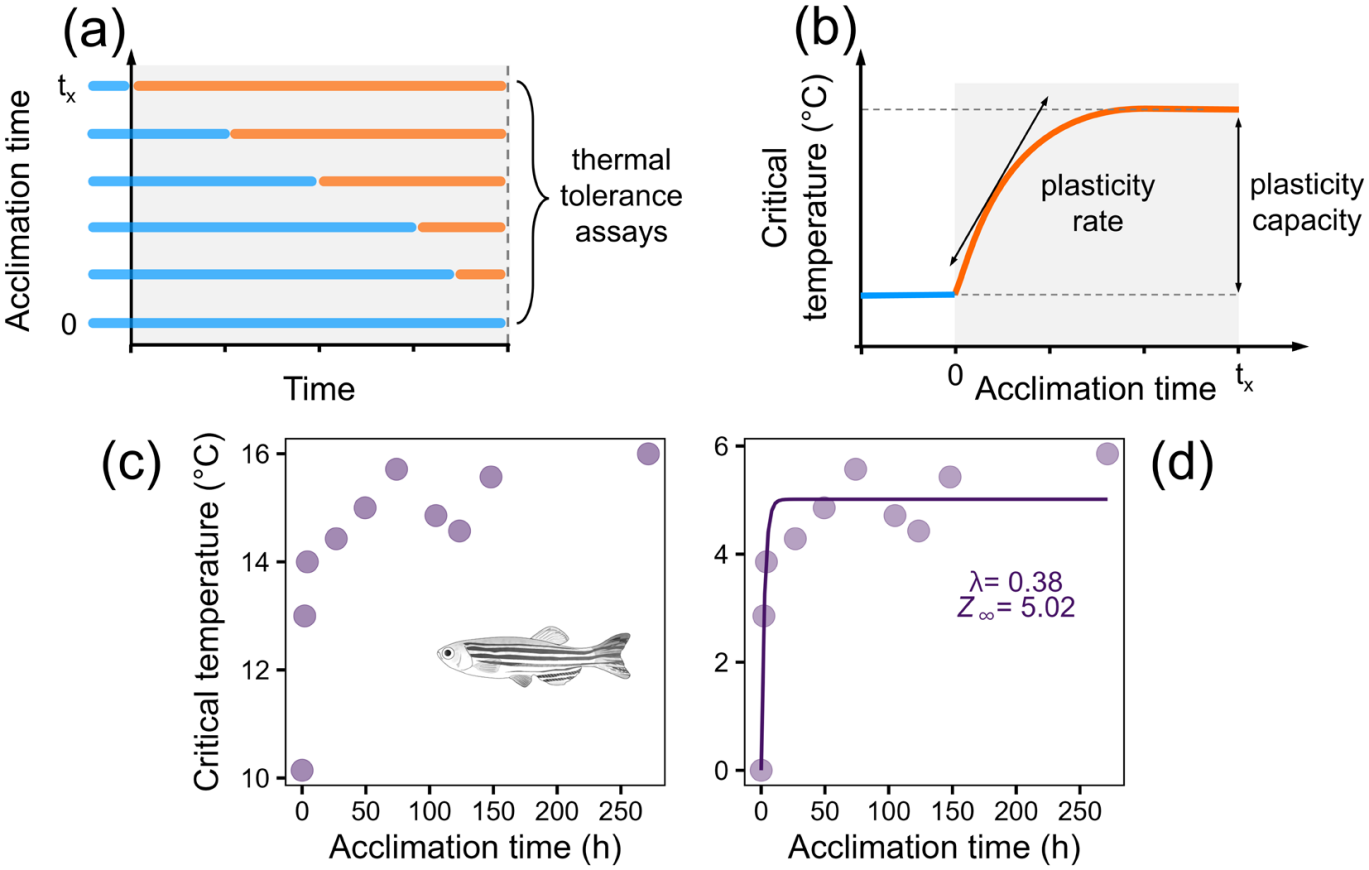
(a) We sought published data from experiments where the study organisms, previously acclimated to an initial temperature (blue lines), were abruptly shifted to a new acclimation temperature (orange lines) at different points in time, creating variation in the amount of time that the new acclimation temperature was experienced before thermal tolerance was measured (dashed line). (b) Conceptual illustration of how the timeseries of measurements resulting from (a) was used to estimate the rate and capacity of plasticity as thermal tolerance changed from the value acclimated to the initial temperature, to the value completely acclimated to the new temperature. (c) Example data set from the fish *Danio rerio* (Aslanidi and Kharakoz 2021) showing the temporal dynamics of plasticity in thermal tolerance. (d) The rate of‐ and capacity for plasticity in thermal tolerance was calculated by first rescaling the measurements of critical temperature to a value of 0 at the first measurement time point and then fitting the model *Z*_*t*_ = *Z*_*∞*_(1‐*e* ^−*λt*^), where *Z*_*∞*_ is the capacity for plasticity (°C) and *λ* (h^−1^) is the rate of plasticity. To make plasticity capacity estimates comparable among experiments that varied in the difference between the initial and new acclimation temperatures, each estimate of plasticity capacity was transformed into an acclimation response ratio (ARR°C °C^−1^, see Methods for further details).

Einum and Burton (2023) provided a method to calculate a single parameter that describes the rate of phenotypic plasticity. In the current analysis, we required estimates of plasticity capacity that corresponded to estimates of the rate of plasticity. To obtain estimates of both plasticity rate and capacity, we first rescaled each experimental data set to have a critical temperature of 0°C at the first measurement timepoint and with a subsequent increase, that is, $Z_{t}=|z_{t}-\bar{z_{0}}|$, where *Z*_*t*_ is the rescaled critical temperature at time t (in hours). We then fitted the model *Z*_*t*_ = *Z*_*∞*_(1‐*e* ^−*λt*^), where *Z*_*∞*_ is the rescaled asymptotic critical temperature when acclimation is complete (i.e., plasticity capacity), and *λ* (h^−1^) is the plasticity rate (Figure 1). The model was fitted using the function *nls_multstart* from the *nls.multstart* package v.1.2.0 (Padfield and Matheson 2020) in R v.4.1.2 (R Core Team 2021), and for each data set we extracted the estimated means and variances of the capacity and rate parameters. The model failed to converge for 10 of the 205 experiments, and these were excluded from further analyses. To make plasticity capacity comparable among experiments that varied in the difference between the initial and new acclimation temperatures, we transformed each *Z*_*∞*_ estimate into an acclimation response ratio, or ARR (e.g., Claussen 1977). The ARR is given as the absolute change in critical temperature per degree difference in temperature between the initial temperature (i.e., the first temperature the experimental organisms are completely acclimated to) and the new acclimation temperature once the plastic response is completed, that is, |*Z*_*∞*_/(acclimation temperature—initial temperature)|. The variance associated with each *Z*_*∞*_ estimate was also divided by the difference between the initial and new acclimation temperatures so that plasticity capacity variances were expressed on the same scale as the ARR values. A conceptual illustration of the experimental data we sought to identify in our literature search and the calculation of the rate and capacity of phenotypic plasticity is provided in Figure 1. In the same figure, we also provide examples of our calculations.

### 2.3 Statistical Analysis of the Relationship Between the Rate Of‐ and Capacity for Phenotypic Plasticity

The data set that we used in the following analyses was obtained from 195 different experiments from 39 studies (84 on amphibians, 3 on reptiles, 47 on fishes, 39 on insects, 18 on crustaceans and 4 on flatworms) representing a total of 72 species (39 amphibians, 2 reptiles, 11 fishes, 13 insects, 5 crustaceans and 2 flatworms). To obtain species‐level estimates of plasticity rate and capacity while accounting for uncertainty (variance) in the estimates of *λ* and *Z*_*∞*_ from each reanalysed experiment, we fitted separate univariate meta‐analytical models to the experiment‐level estimates of plasticity rate and plasticity capacity using the function rma.mv in the R package metafor v.3.8‐1 (Viechtbauer 2010). These data have multiple levels of variance, such that a full model should ideally include variance due to study (some studies had several experiments), nonphylogenetic and phylogenetic species‐level variances, as well as experiment‐level variance. However, concerns have been raised regarding the ability of such models to separately estimate the species‐level and study‐level variance components if many of the studies only provide data from one species or data from closely related species (Cinar, Nakagawa, and Viechtbauer 2022). For our data, 30 of the 39 studies only contained data from a single species, and the remaining studies were indeed studying closely related species (e.g., two of the studies provided data from 11 and 17 of the amphibian species, respectively). Thus, we did not include a study effect. The second question one should consider in such analyses is whether one should estimate both the nonphylogenetic and phylogenetic species‐level variances. Simulation studies have shown that usually including both is preferable, but that this may not be the case if phylogenetic relationships among the species in the data (i.e., the mean of the off‐diagonal values in the relatedness matrix) are weak (Cinar, Nakagawa, and Viechtbauer 2022). We created such a matrix based on a tree built using the package rotl v.3.0.12 (Michonneau, Brown, and Winter 2016) and the Open Tree of Life (OpenTreeOfLife et al. 2019) which was made ultrametric using the function ‘compute.brlen’ in package ape v.5.6‐2 (Paradis and Schliep 2018). This tree was then used to compute the species relatedness variance–covariance matrix. The mean of the off‐diagonal values of the matrix was 0.33. Cinar, Nakagawa, and Viechtbauer (2022) suggested that values less than 0.2 makes the nonphylogenetic and phylogenetic variance estimates unreliable. The problem arising under weak phylogenetic relationships is that it becomes arbitrary how the total variance is distributed into these two components (Cinar, Nakagawa, and Viechtbauer 2022). In our initial modelling we may have observed such an effect, where the majority of the species‐level variance was assigned to the phylogenetic component for the rate of plasticity model, whereas it was assigned to the nonphylogenetic component for the capacity for plasticity model. Thus, the models presented here only estimate the nonphylogenetic species‐level variance, together with the experiment‐level variance.

Our previous work identified an effect of acclimation temperature on plasticity rates (Einum and Burton 2023), and thus in the full models this was included as a fixed effect. Acclimation temperatures experienced were similar across the taxonomical classes (mean °C [range], amphibians: 20 [2–38], reptiles 34 [27–40], fish 21 [4–35], insects 20 [4–37], crustaceans 18 [4–30], flatworms 15 [5–25]). As in previous analyses, we also included the possibility for an effect of trait type (i.e., CTmax or CTmin) on the experiment‐level estimates of plasticity rate and capacity. The estimation procedure for the rate used here differs from the one we used previously (Einum and Burton 2023) in that it does not assume that complete acclimation is reached during the experiment, and thus we do not have to control for bias due to incomplete acclimation as we have done previously. We compared the support for models with or without temperature and/or trait type effects for both traits (rate and capacity) using AICc. We present the estimated model parameters from the model receiving the strongest support (refitted using REML) as well as heterogeneity *I* ^*2*^ and pseudo‐*R* ^*2*^ values. For each of the fitted models, inspection of residual plots indicated that assumptions of their normality and homogeneity were satisfied. Based on the best models for rate and capacity, we extracted the species‐level random effect coefficients and tested for a correlation between these. Finally, we evaluated how the species‐level estimates of plasticity rate and capacity varied with respect to taxonomic class. All statistical analyses were conducted in R version 4.2.0 (R Development Core Team 2023).

## 3 Results

Inspection of model predictions fitted against observed values suggests that the temporal dynamics of thermal tolerance is well described by the applied model (Figure S1). Overall median estimated values of lambda and ARR were 0.026 h^−1^ and 0.24 °C °C^−1^, respectively. For the meta‐analysis of lambda, a model that included the effects of temperature and trait type was considerably better than those with only one or neither of them (AICc = −512.1 with temperature and trait type, −506.1 with temperature, −496.4 with trait type, −491.1 with neither). Lambda increased with increasing temperature and was lower for CTmin than for CTmax measurements (Table 1). The mean predicted rate of plasticity (calculated for CTmax traits) increased from 0.020 to 0.058 h^−1^ between the minimum and maximum acclimation temperatures present in our data (1.8°C–40°C). For the meta‐analysis of ARR, there was evidence for an effect of trait type, but not acclimation temperature (AICc = −28.0 with trait type, −27.4 with both temperature and trait type, −23.7 with temperature, −24.4 with neither). ARR was higher for CTmin than for CTmax measurements. Species‐effects accounted for 22 and 65% of the total variation in lambda and ARR, respectively (Table 1).

**TABLE 1:** Summary of the best‐fitting meta‐analytical models describing variation in the rate of‐ and capacity for plasticity in thermal tolerance among different species of ectothermic animals.

Fixed effects	Estimate	95% CI
Rate of plasticity (*λ*)		
Intercept (CTmax)	0.0178	0.0070 to 0.0285
Acclimation temperature	0.0010	0.0006 to 0.0015
Trait type, CTmin	−0.0158	−0.0263 to −0.0053

Random effects	SD	*I* ^2^
Species	0.0148	21.79
Experiment	0.0231	78.01

Fixed effects	Estimate	95% CI
Capacity for plasticity (ARR)		
Intercept (CTmax)	0.2112	0.1824–0.2401
Trait type, CTmin	0.0518	0.0099–0.0937

Random effects	SD	*I* ^2^
Species	0.0930	65.42
Experiment	0.0672	34.11

*Note:* In each model, the effect of trait measurement type is presented so that the effect of CTmin measurements is evaluated relative to the effect of CTmax measurements (i.e., the intercept). The amount of variance (*I* ^2^, %) explained by the respective random effects are given. Total *I* ^2^ for lambda and ARR was 99.8% and 99.5%, respectively. Pseudo‐R2 was 18.0 and 6.5% for the plasticity rate and capacity models, respectively.

**FIGURE 2:**
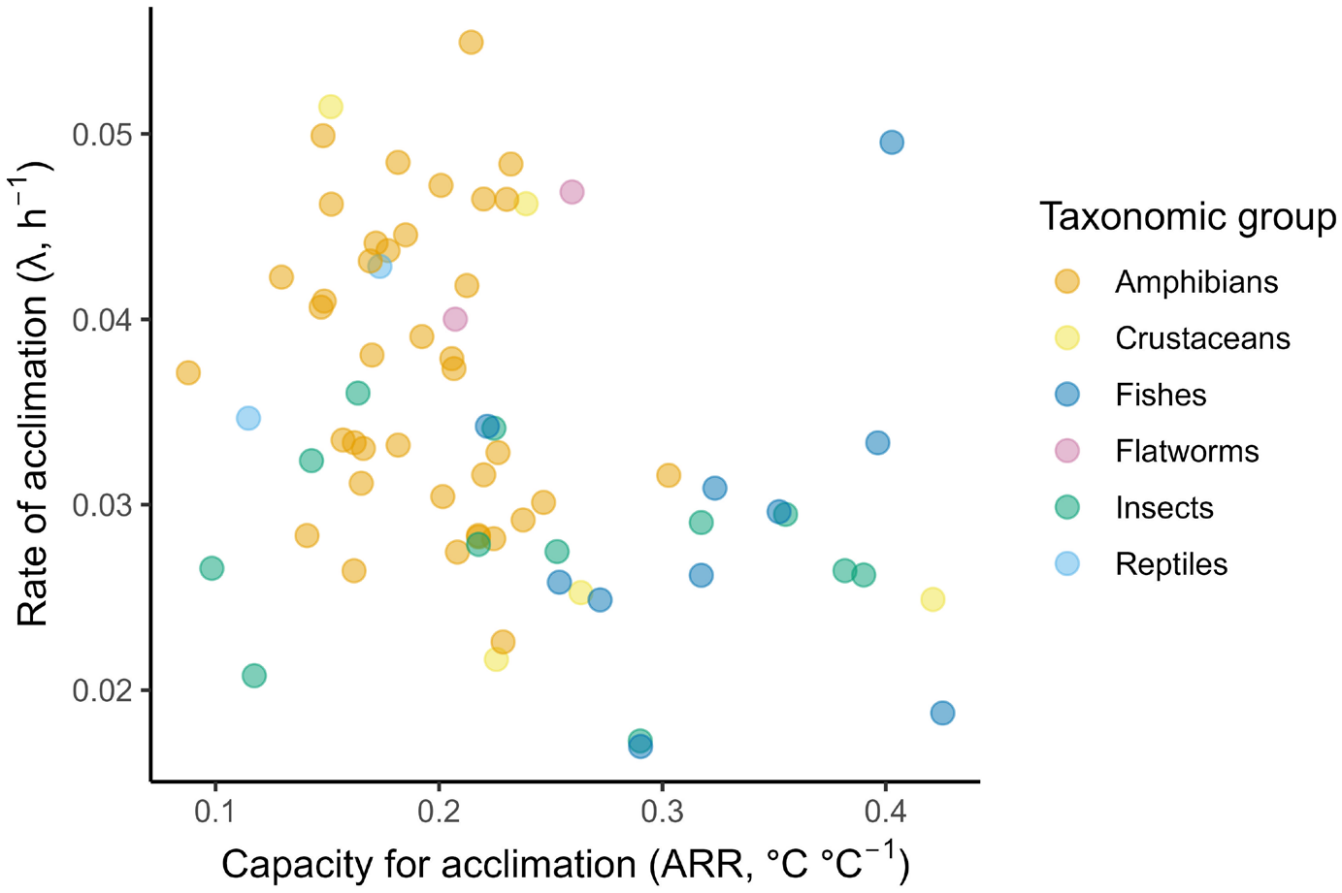
Relationship between the rate of‐ and capacity for plasticity in thermal tolerance of ectothermic animals. Each data point represents an estimate for a different species (*n* = 72). These species‐level estimates were extracted from meta‐analytical regression models applied to estimates of the rate of‐ and capacity for plasticity in thermal tolerance that were obtained by reanalysing published experimental data sets. Given that some species featured in several experiments, the species‐level estimates of plasticity rate and capacity are a synthesis of a larger sample of experiment‐level estimates (*n* = 195). Species‐level estimates for plasticity rate are standardised to a common acclimation temperature of 20°C (temperature influences plasticity rate, but not capacity, see Table 1 for a summary of the meta‐analytical regression models that were applied to our estimates of plasticity rate and capacity).

**FIGURE 3:**
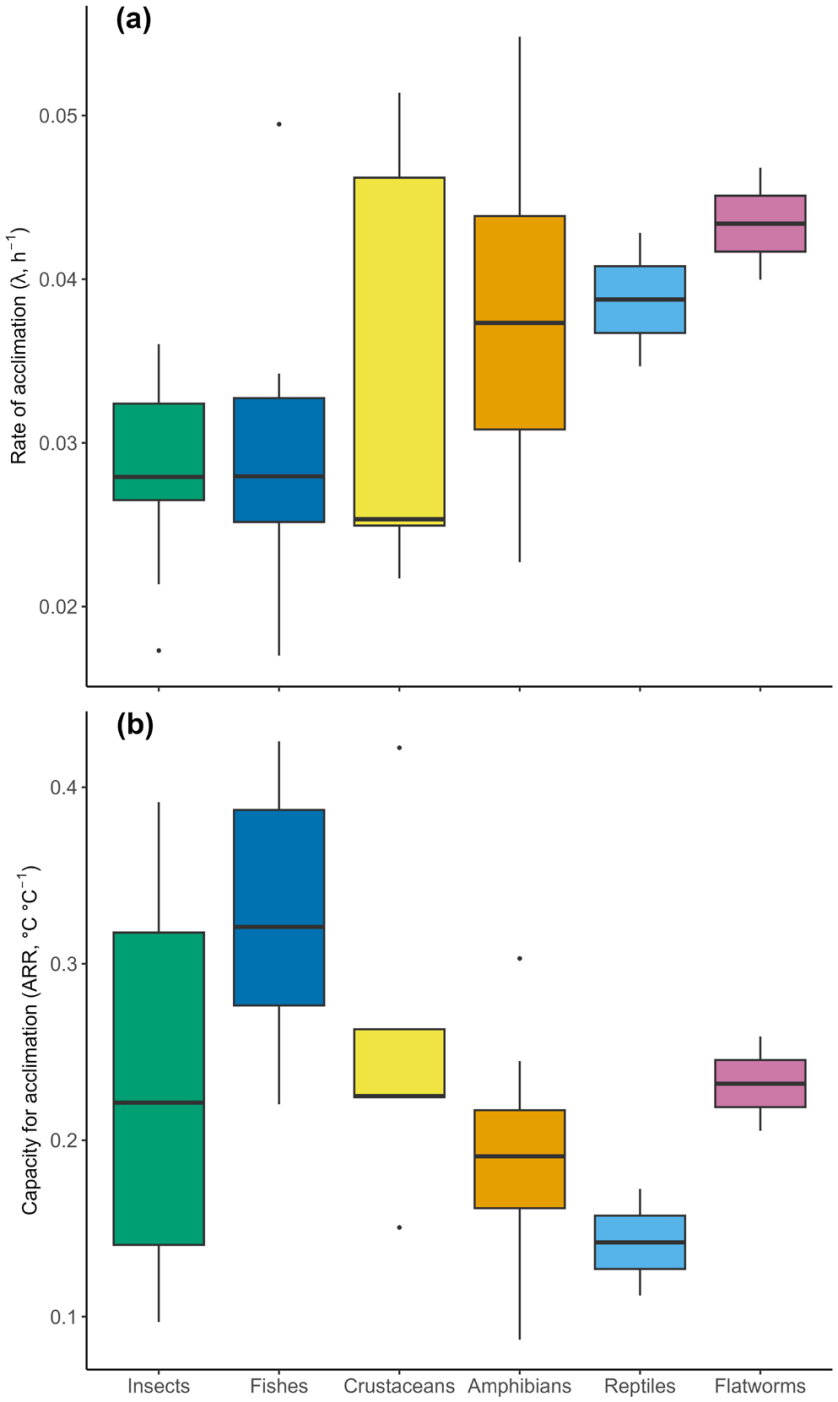
Species‐level estimates of the (a) rate of‐ and (b) capacity for plasticity in thermal tolerance of ectothermic animals. Data are the same as those shown in Figure 2 but are replotted according to taxonomic class. Estimates for plasticity rate are standardised to a common acclimation temperature of 20°C. Boxes represent the 25th and 75th percentiles, and whiskers represent 1.5 interquartile range from the box.

Species‐specific estimates of lambda (standardised to 20°C for CTmax) and ARR (for CTmax) obtained from the meta‐analyses ranged from 0.0220–0.0525 h^−1^ and 0.0860°C–0.4200°C °C^−1^, respectively. The bivariate distribution of these two traits deviated somewhat from normality (Figure 2), but they were negatively correlated independent of this assumption (Pearson's correlation = −0.29, *p* = 0.014, Spearman correlation = −0.31, *p* = 0.009, *n* = 72). Although our approach to estimate the rate of plasticity differed from a previous analysis (Einum and Burton 2023), and the experiments that were included in the current analysis differed due to changes in selection criteria (in particular, the current analyses did not exclude studies that had high uncertainty in the estimated lambda, see Methods), the taxonomic pattern of variation in rate of plasticity remained very similar, with the fastest rates of plasticity being observed among reptiles and amphibia, and the slowest rates among fish, crustaceans and insects (Figure 3a). Flatworms were not included in the previous analysis, and the two species included in the current analysis had relatively high estimates of plasticity. The overall negative correlation between rate and capacity for plasticity were mirrored by a tendency for the taxa with the slowest rates of plasticity (fish, crustaceans and insects) to have a higher capacity for plasticity in thermal tolerance (Figure 3b).

## 4 Discussion

By adopting a comparative approach, we aim to enhance our understanding of how phenotypic plasticity has evolved to facilitate organismal resilience to environmental change. The observed negative correlation between the plasticity rate and capacity was not consistent with expectations that can be inferred from current theory which seeks to understand the conditions under which phenotypic plasticity can evolve (Lande 2014; Siljestam and Östman 2017). For example, Siljestam and Östman (2017) consider the effect of the rate of environmental change relative to the rate of plasticity on the evolution of the capacity component of plasticity. In their model, a fast rate of environmental change, relative to the rate of plasticity, results in the evolution of a low capacity for plasticity. This might be interpreted as suggesting that for a given pattern of environmental fluctuation, higher rates of plasticity (and hence slower relative rates of environmental change) would select for a higher capacity and thus that a positive correlation between rate and capacity would emerge. A similar prediction can be derived from Lande (2014). A key outcome of his model is that more predictable environments will select for a larger capacity for plasticity. A less appreciated feature of this work is that environmental predictability depends on the rate of plasticity. Specifically, for a given level of environmental autocorrelation, environmental predictability should be averaged over development time, and thus a high rate of plasticity (and short development time) will give a higher predictability, and thus a higher capacity. One might then also interpret this as predicting a positive correlation between the rate and capacity of phenotypic plasticity. However, the main focus of both Siljestam and Östman (2017) and Lande (2014) is to make predictions regarding the evolution of plasticity capacity. Neither consider the scenario that the rate of plasticity might also evolve in response to the pattern of fluctuations in the environment. This mismatch between theory and data suggests that further theoretical treatment of this topic may yield insight into the relationship between plasticity rate and plasticity capacity observed here. Specifically, there is a need to develop models that allow for simultaneous evolution of both rates and capacities, as this can be used to assess how these should co‐evolve under contrasting patterns of environmental variation. At present, this can only be examined verbally, where one might argue that rapid unpredictable variation could simultaneously select for a high rate to avoid the phenotype to lag behind the optimum and at the same time that such variation reduces predictability and hence selects for a lower capacity of plasticity (Lande 2014). Such a prediction would match the observations of the current study. Yet, it remains to be evaluated whether such negative correlations actually arise from co‐evolution in more formal models. Finally, although we confront our observations with theoretical predictions that investigate adaptive outcomes, an alternative nonadaptive interpretation of the pattern observed here is that a plasticity response of high capacity simply requires more time to produce and therefore plasticity is unable to be realised rapidly. Experimental studies that assess the adaptive value of covariance between the rate and capacity of plasticity may give further insights into this outstanding question.

The negative relationship between plasticity rate and plasticity capacity revealed in our analysis also provides some insight into the costs that are presumed to influence the evolution of phenotypic plasticity. Plasticity costs can be categorised into costs of maintenance and production (Auld, Agrawal, and Relyea 2010). For maintenance costs, more plastic genotypes must invest more in maintaining the ‘machinery’ needed to respond to the environment. This cost is paid in all environments. Conversely, production costs are only paid when actually changing the phenotype and these are presumably outweighed by the benefits provided by the plasticity. Hence, maintenance costs are expected to constrain the evolution of plasticity to a greater extent than production costs (Sultan and Spencer 2002). We have previously proposed that the cost of having plasticity that can occur rapidly may be as high, if not higher, than the cost of having plasticity that can occur in large magnitude (Burton, Ratikainen, and Einum 2022; Einum and Burton 2023). Our current analysis provides indirect support for this hypothesis. Specifically, in the absence of such costs there would be no disadvantage associated with maintaining the ability to implement plasticity rapidly in stable environments. Accordingly then, one might anticipate there to be no clear relationship between plasticity rate and plasticity capacity as the rate component of plasticity would be free to evolve independently of the costs that are presumed to limit evolution of the capacity component in stable environments (van Buskirk and Steiner 2009; Auld, Agrawal, and Relyea 2010).

The evolution of plasticity has been put forward as a means by which organisms might cope with novel patterns of environmental change such as those brought about by climate change (Chevin, Lande, and Mace 2010). Our results, however, indicate that if those patterns include changes in the environment that are simultaneously rapid, large in amplitude and unpredictable, such as the unprecedented shifts in temperature that are already affecting natural populations (Cerrano et al. 2000; Campbell‐Staton et al. 2017; Genin et al. 2020) and which are predicted to become more frequent (Coumou and Rahmstorf 2012; Fischer, Sippel, and Knutti 2021), it may be difficult for organisms to evolve plasticity in thermal tolerance that can occur rapidly enough and with sufficient capacity to mitigate them. Our results also indicate that the effects of rapid environmental change might not manifest evenly across the tree of life. Rates of plasticity in thermal tolerance were higher in reptilians and amphibians than in fishes, crustaceans and insects. A similar phylogenetic pattern was evident in a previous analysis (see figure 2 in Einum and Burton 2023), which begs the question of why has such variation in the rate of plasticity in thermal tolerance evolved? We previously (Einum and Burton 2023) speculated that the observed trend in plasticity rate results from adaptation to different patterns of environmental variation, because the groups showing most rapid plasticity (the reptiles and amphibians) tend to inhabit more thermally variable terrestrial or semiaquatic environments, whereas two of the remaining groups with slowest plasticity (the fishes and crustaceans) tend to be found in the more stable aquatic realm. However, this observed relationship between plasticity rate and environmental variation (i.e., habitat use in this case) is confounded with phylogeny. Furthermore, the two species of flatworms included in the current analysis are also aquatic, and yet show the highest rates of plasticity. Thus, it remains an outstanding question as to whether rates of plasticity evolve in response to different patterns of environmental variation. However, this question could be addressed in experimental work that targets populations or species that experience known and contrasting patterns of environmental variability.

Currently, the manner in which rates of phenotypic plasticity are modulated by pervasive characteristics of the environment, such as ambient temperature, is largely unknown. Our analysis revealed that in ectothermic animals, plastic adjustments of the phenotype occur more rapidly at warmer acclimation temperatures, whereas the capacity shows no such response. In mechanistic terms, the relationship between plasticity rate and acclimation temperature may be attributed to the general effect of temperature on biochemical rates (Brown et al. 2004). We emphasise that this relationship results from a comparison of species likely adapted to different temperatures. The data analysed here originate from experiments that were likely performed at acclimation temperatures within the typical range that each species experiences in the wild. This means that acclimation to high temperature was more likely to be measured in species living in warmer climates, and *vice versa*. Thus, it remains unclear if the relationship between acclimation temperature and the rate of plasticity is causative, though it is consistent with experimental data from a single species whereby acclimation to high temperature proceeded more rapidly than acclimation to low temperature (Burton, Lakka, and Einum 2020).

An assumption in our analysis is that our estimates of plasticity in whole organism thermal tolerance represent cases of plasticity that are predominantly active rather than passive. The distinction between active and passive plasticity is important because active plasticity responses are perceived as being adaptive (i.e., have been shaped by selection and result in better matching between the phenotype and environment, Ghalambor et al. 2007), reflecting often highly integrated phenotypic changes that can occur across different levels of organisation (Forsman 2015; Havird et al. 2020). Indeed, plasticity in whole organism thermal tolerance is associated with change in lower‐order traits that require active regulation, for example, the expression of heat‐shock proteins or production of haemoglobin (Seidl, Pirow, and Paul 2005; Tomanek 2008). On the other hand, passive plasticity responses are not actively regulated by the organism and represent an unavoidable consequence of the environment (Forsman 2015; Havird et al. 2020). For example, many physiological rates increase with temperature, solely due to molecular thermodynamics (Arrhenius 1915). Thus, given that thermal tolerance tends to display such a response to temperature we cannot exclude the possibility that our estimates of plasticity include some contributions from passive plasticity.

In conclusion, our study provides a novel approach to simultaneously estimate the rate and capacity of plasticity from plasticity time course data. By employing meta‐analytical methods which enabled us to account for uncertainties in our estimates of plasticity rate and capacity as well as the effects of acclimation temperature, we show that the rate of‐ and capacity for plasticity is negatively correlated at a species level. However, we urge caution in the generalisation of this observation given that the taxonomic coverage and sample size of our analysis is relatively modest. Accordingly, we encourage more empirical and theoretical focus on the rate component of plasticity as this will likely yield novel insights into how phenotypic plasticity facilitates biological resilience under rapid environmental change.

## Author Contributions

T.B. and S.E. designed research. T.B. and S.E. performed research; S.E. analysed data with input from T.B. and T.B. wrote the paper with input from S.E.

### Peer Review

The peer review history for this article is available at https://www.webofscience.com/api/gateway/wos/peer‐review/10.1111/ele.70046.

## Supporting information


**Figure S1.** Phenotypic plasticity in thermal tolerance (measured as °C change following transfer to a new temperature) as a function of acclimation duration in individual experiments. Data are rescaled so that the critical temperature is 0 °C at the first measurement timepoint and fitted with the model *Z*_*t*_ = *Z*_*∞*_(1‐*e* ^−*λt*^), where *Z*_*∞*_ is the asymptotic critical temperature when acclimation is complete (i.e., plasticity capacity), and *λ* (h^−1^) is the plasticity rate.

## Data Availability

Data and code are available at Dryad (https://doi.org/10.5061/dryad.18931zd4v).
