# Supplementary material for: High Capacity for Physiological Plasticity Occurs at a Slow Rate in Ectotherms
Source: Ecol Lett. 2025 Jan 20;28(1):e70046. doi: 10.1111/ele.70046 (PMC11744337; doi:10.1111/ele.70046)
Supplement: Supplementary file 1 — Figure S1. Phenotypic plasticity in thermal tolerance (measured as °C change following transfer to a new temperature) as a function of acclimation duration in individual experiments. Data are rescaled so that the critical temperature is 0 °C at the first measurement timepoint and fitted with the model Z t = Z ∞ (1‐e −λt ), where Z ∞ is the asymptotic critical temperature when acclimation is complete (i.e., plasticity capacity), and λ (h−1) is the plasticity rate. [file ELE-28-0-s001.docx]

Supporting Information for:

High capacity for physiological plasticity occurs slowly in ectotherms

Tim Burton^1*^ and Sigurd Einum^2^

1. Norwegian Institute for Nature Research, Høgskoleringen 9, 7034, Trondheim, Norway, tim.burton@nina.no
2. Centre for Biodiversity Dynamics, Department of Biology, Norwegian University of Science and Technology, Realfagbygget, NO-7491 Trondheim, Norway, sigurd.einum@ntnu.no

*corresponding author

**This file includes:**

Supplementary figure S1

**Fig. S1.** Phenotypic plasticity in thermal tolerance (measured as °C change following transfer to a new temperature) as a function of acclimation duration in individual experiments. Data are rescaled so that the critical temperature is 0°C at the first measurement timepoint and fitted with the model *Z_t_* = *Z_∞_*(1-*e*^-^*^λt^*), where *Z_∞_* is the asymptotic critical temperature when acclimation is complete (i.e. plasticity capacity), and *λ* (h^-1^) is the plasticity rate.

**
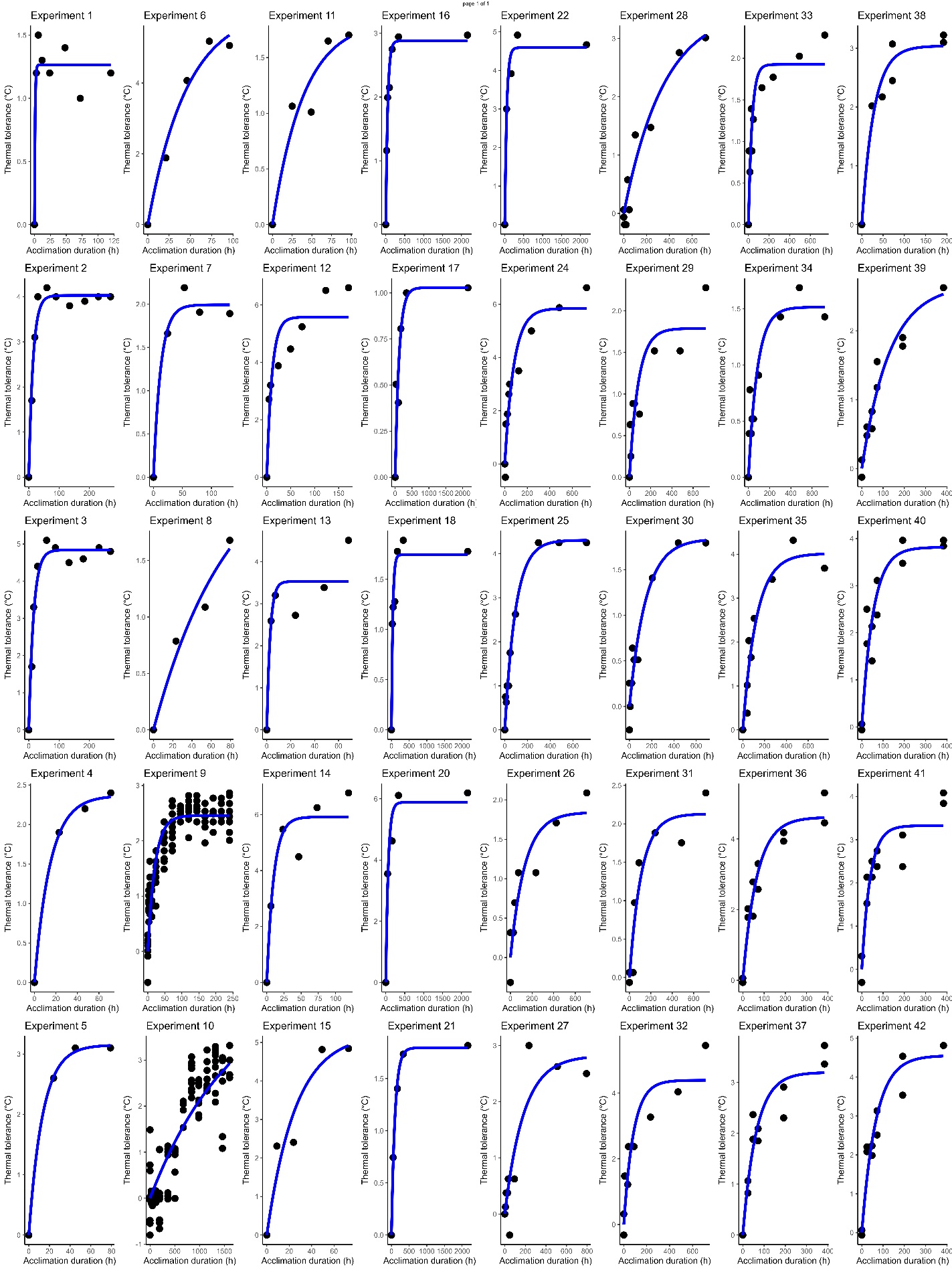
**

**Fig. S1**


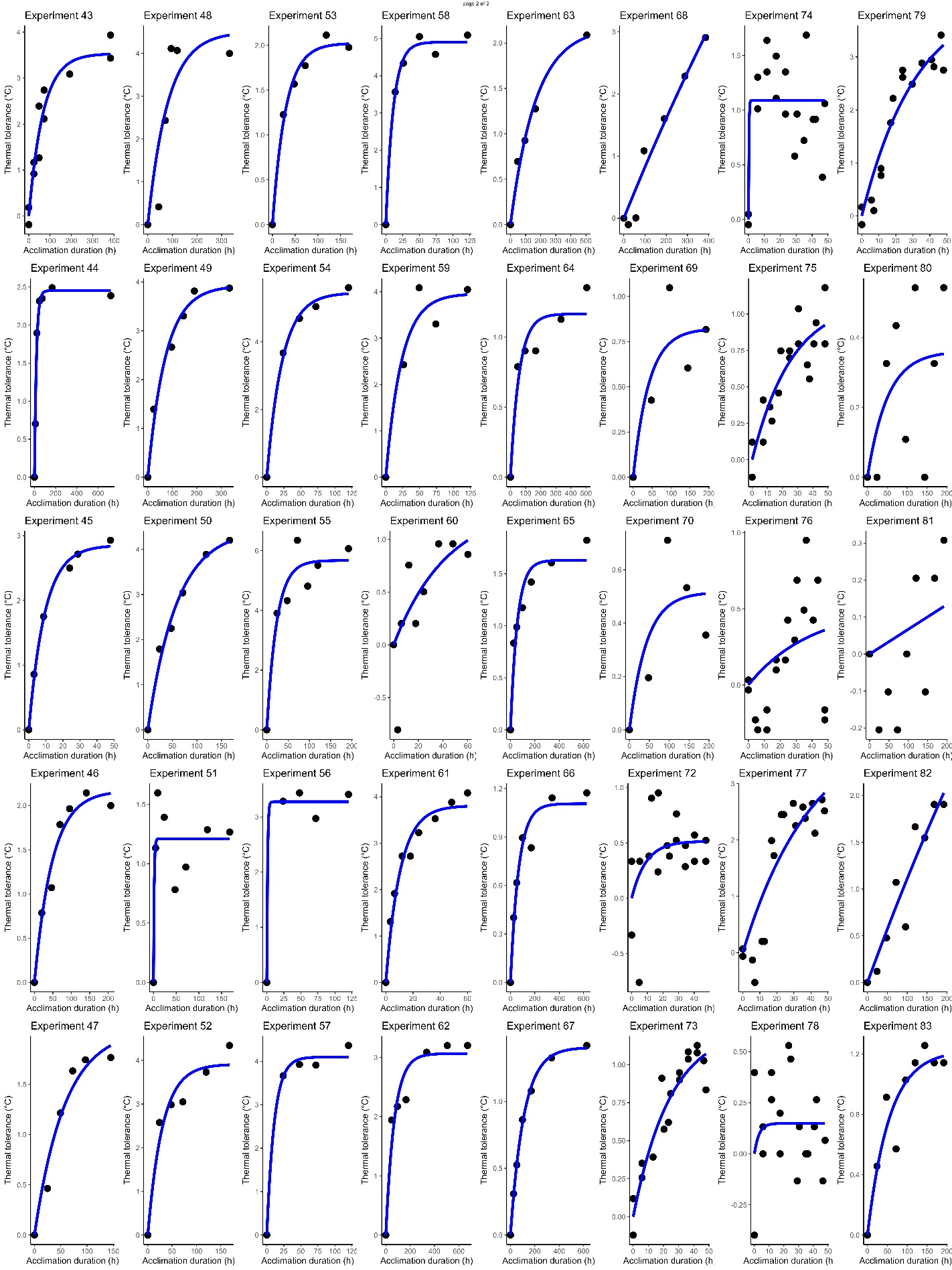


**Fig. S1**. continued


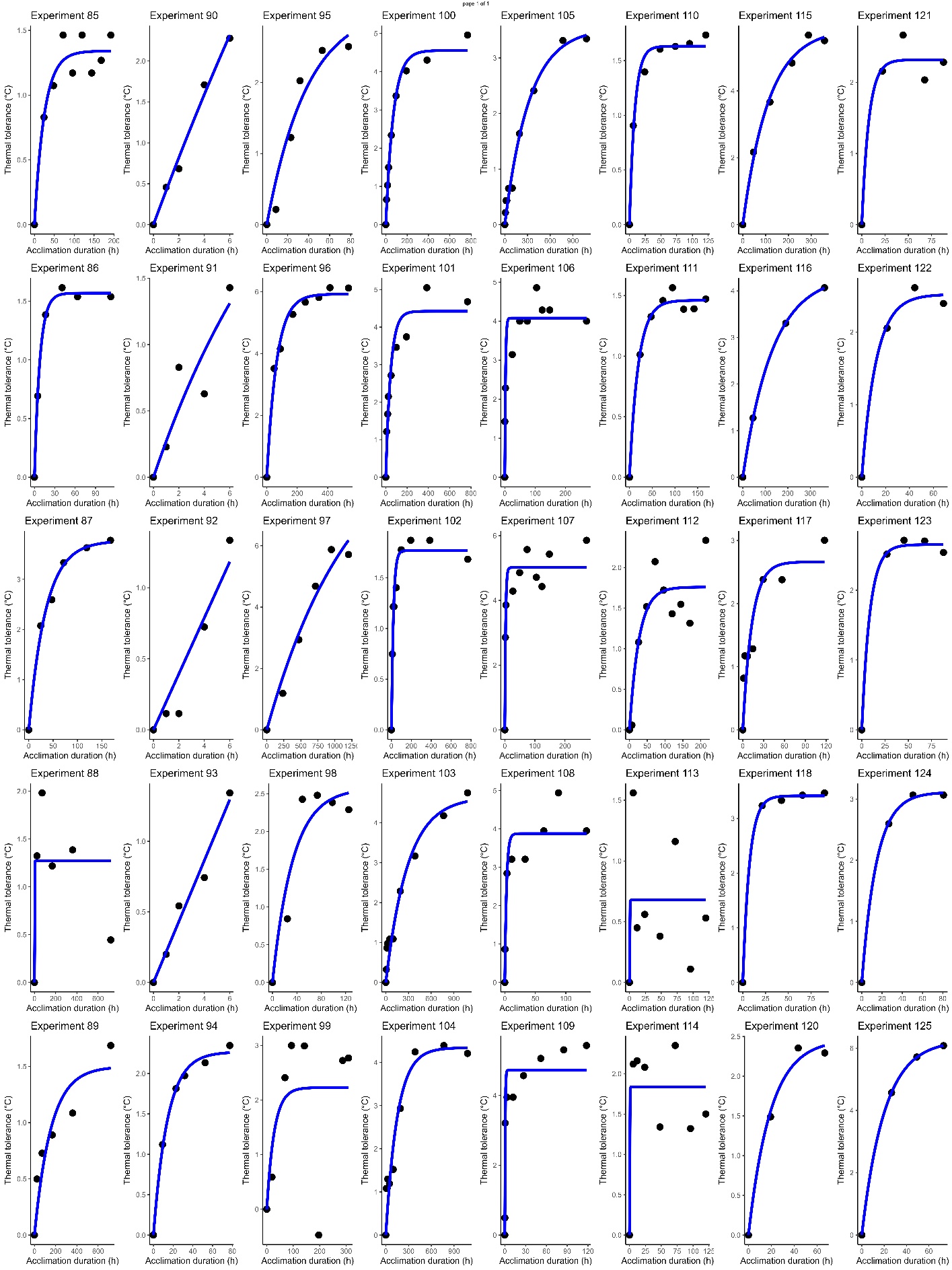


**Fig. S1**. continued


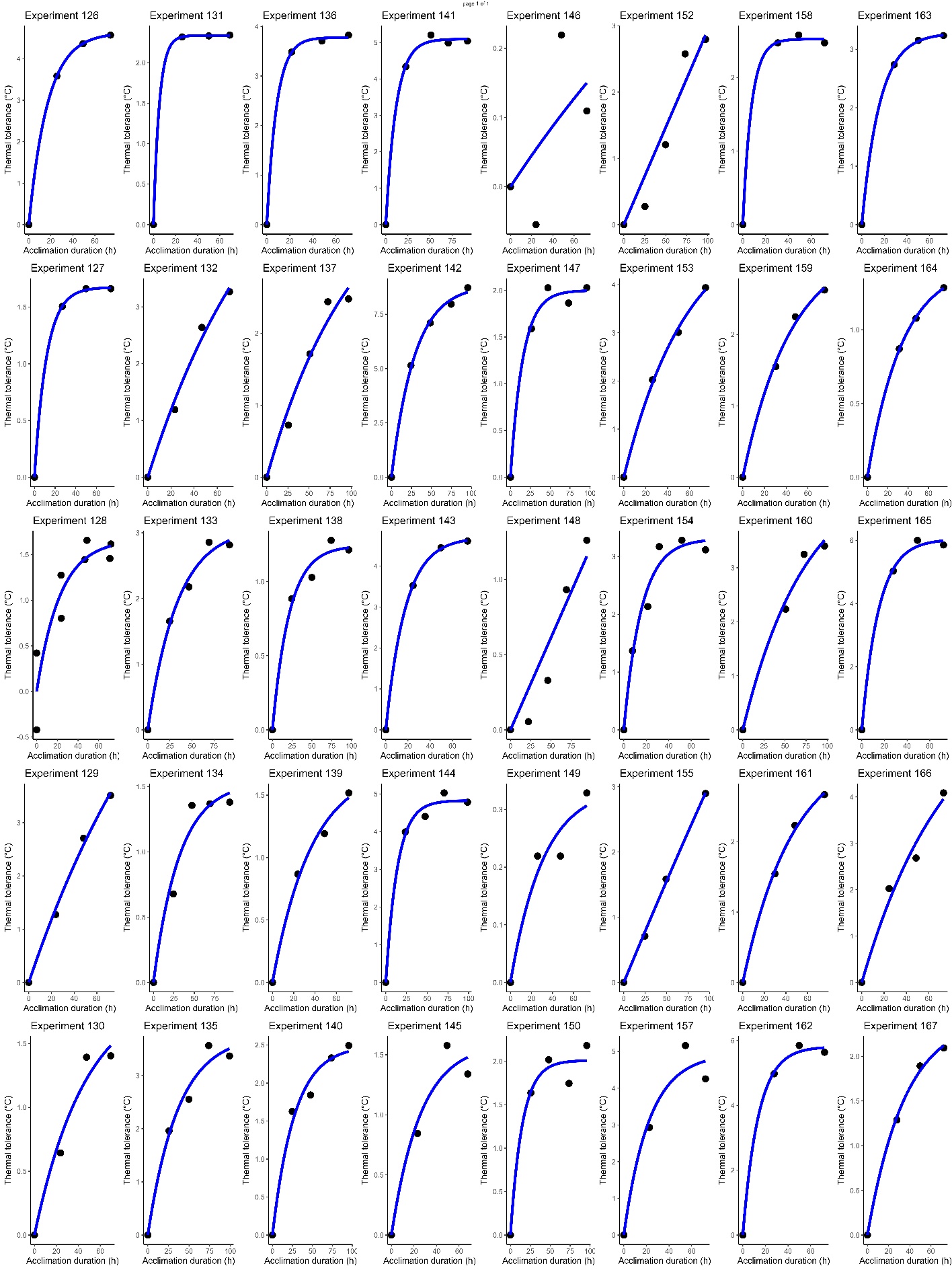


**Fig. S1**. continued


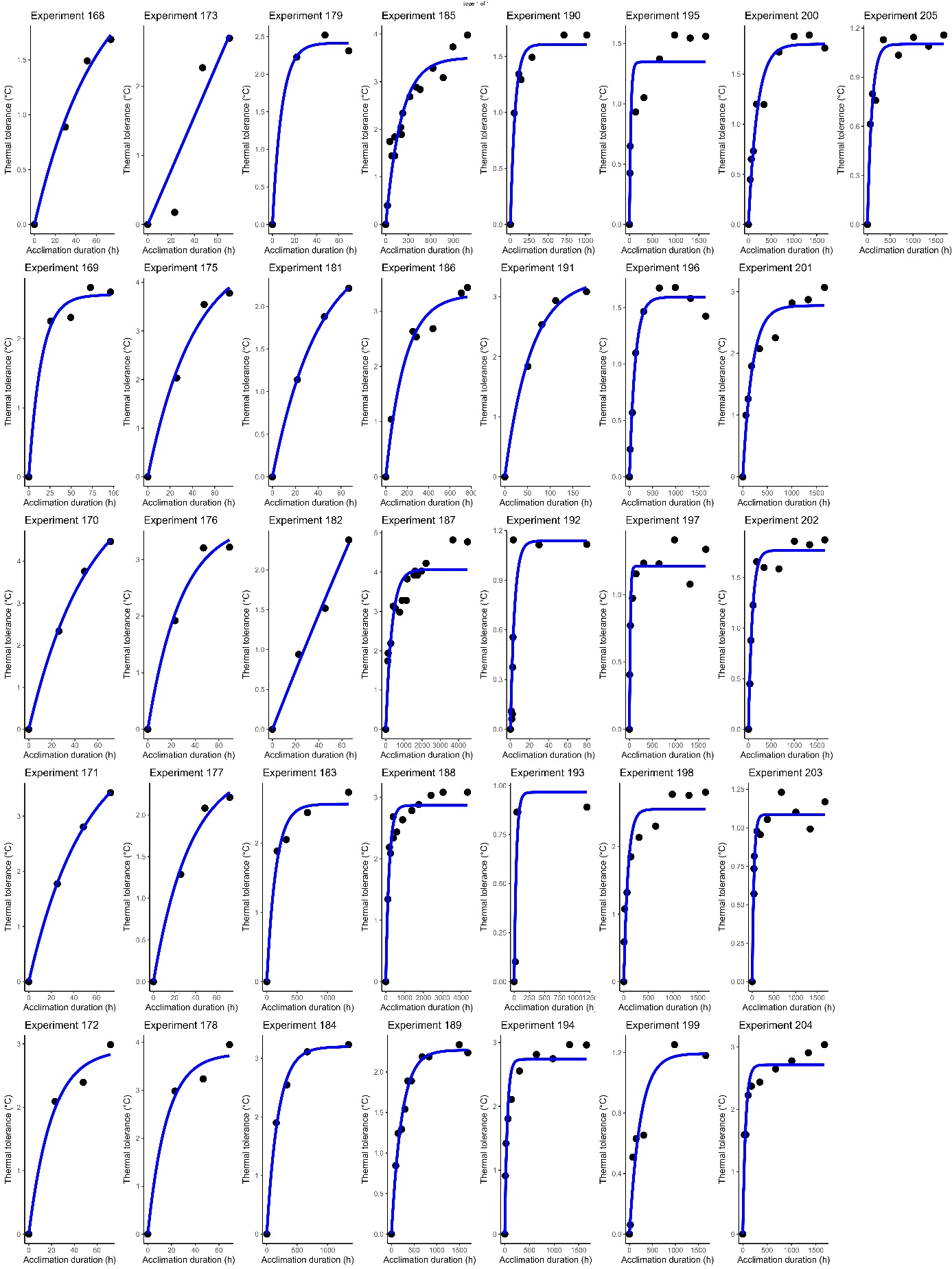


**Fig. S1**. continued
